# Morphology and N_2_ Permeance of Sputtered Pd-Ag Ultra-Thin Film Membranes

**DOI:** 10.3390/molecules21020210

**Published:** 2016-02-10

**Authors:** Ekain Fernandez, Jose Angel Sanchez-Garcia, Jose Luis Viviente, Martin van Sint Annaland, Fausto Gallucci, David A. Pacheco Tanaka

**Affiliations:** 1 Energy and Environment Division, TECNALIA, Mikeletegi Pasealekua 2, 20009 San Sebastián-Donostia, Spain; jangel.sanchez@tecnalia.com (J.A.S.-G.); joseluis.viviente@tecnalia.com (J.L.V.); 2 Chemical Process Intensification, Department of Chemical Engineering and Chemistry, Eindhoven University of Technology, De Rondom 70 5612 AZ Eindhoven, The Netherlands; M.v.SintAnnaland@tue.nl (M.S.A.); F.Gallucci@tue.nl (F.G.)

**Keywords:** palladium-silver alloy, PVD magnetron sputtering, ultra-thin film, H_2_ separation, film growth mechanisms

## Abstract

The influence of the temperature during the growth of Pd-Ag films by PVD magnetron sputtering onto polished silicon wafers was studied in order to avoid the effect of the support roughness on the layer growth. The surfaces of the Pd-Ag membrane films were analyzed by atomic force microscopy (AFM), and the results indicate an increase of the grain size from 120 to 250–270 nm and film surface roughness from 4–5 to 10–12 nm when increasing the temperature from around 360–510 K. After selecting the conditions for obtaining the smallest grain size onto silicon wafer, thin Pd-Ag (0.5–2 µm thick) films were deposited onto different types of porous supports to study the influence of the porous support, layer thickness and target power on the selective layer microstructure and membrane properties. The Pd-Ag layers deposited onto ZrO_2_ 3-nm top layer supports (smallest pore size among all tested) present high N_2_ permeance in the order of 10^−6^ mol·m^−^^2^·s^−^^1^·Pa^−^^1^ at room temperature.

## 1. Introduction

In recent years, palladium membranes have received growing interest for hydrogen purification (and H_2_ production by membrane reactors) due to their extremely high permeability and selectivity towards H_2_. However, pure Pd membranes are often damaged by hydrogen embrittlement, as a result of the α-β phase transition of palladium hydride, which may occur below the critical temperature (293 °C) and pressure (2 MPa) [1]. To avoid embrittlement, Pd is often alloyed with Ag, with the additional benefit of higher H_2_ permeability up to 70% compared to pure Pd. The optimal value for the hydrogen permeability is reached with a silver content around 23 wt.% Ag (Pd_77_Ag_23_) [2,3]. Most of the commercially-available palladium-alloy membranes are above 20–30 microns thick and often self-supported [4,5], and therefore, they have low hydrogen permeation flux (due to the larger thickness) and higher costs (due to the high Pd content and high membrane area required). In order to overcome these limitations, very thin Pd films (<5 µm) deposited onto porous supports are required. There are several technologies reported in the literature for deposition of Pd and Pd-alloy layers onto supports, such as physical vapor deposition (PVD), including magnetron sputtering (PVD-MS), chemical vapor deposition (CVD), electroless plating (ELP) and electroplating (EP) [6]. PVD-MS is a very attractive deposition technique, because it could provide sub-micrometric uniform layers with a controlled composition across the thickness, with required Ag contents up to or higher than 23%, which is difficult by the ELP technique [7]. Other important advantages of PVD-MS are its waste liquid-free operation [8] and the versatility for binary, ternary and multi-component Pd-alloy film deposition [9].

SINTEF manufactures self-supported membranes by PVD-MS in two steps. First, films are prepared by sputtering from a Pd/Ag23 wt.% target onto polished silicon single-crystal substrates, then the films are carefully lifted off from the silicon substrate and mounted in a self-supported configuration [10,11]. Compared to the two-step method, the direct PVD-MS deposition of Pd-based layers onto porous supports is an attractive alternative, since it is less complicated (only one step), less expensive, faster and easier to apply in a module.

The effect of the PVD process parameters on the morphology of the deposited Pd-based film and on the permeation properties has been reported in the literature and is briefly reviewed here. In the first studies, mainly SEM, XRD and gas permeation were used for the analysis of the grain size of the grown layers and for the detection and quantification of pinholes. Jayaraman *et al.* deposited ultrathin Pd films on porous ceramic supports by RF magnetron sputtering and studied the influence of the support temperature and surface roughness on the structure of the deposited film by SEM and XRD [12]; it was observed that the grain size of the Pd film increases by increasing the substrate temperature and the N_2_ gas tightness increases by decreasing the surface roughness of the ceramic support. The optimum coating temperature was 673 K at which 30 nm-sized Pd grains were produced: at this temperature, a good adhesion was observed between the γ-alumina support and the Pd layer up to thicknesses of 300 nm. The N_2_ permeance of the ultra-thin membranes was 5.4 × 10^−7^ mol·m^−2^·s^−1^·Pa^−1^ at room temperature [13], very far from the target of a N_2_ permeance of <1.0 × 10^−9^ mol·m^−2^·s^−1^·Pa^−1^ in order to obtain a high purity hydrogen stream. At greater thickness, the delamination of the layers from the support was observed probably due to lattice stresses involved in the deposition and cooling process. Zhao *et al.* deposited Pd and Pd-Ag membranes with a thickness <1 µm by PVD-MS on commercial microfiltration membranes coated with a γ-Al_2_O_3_ layer using a Pd-24 wt.% Ag alloy as the target material [14]. The effect of the substrate temperature (varied from 300–450 °C) on the helium permeance was studied obtaining the lowest permeance between 350 and 400 °C with a value in the order of 10^−7^ mol·m^−2^·s^−1^·Pa^−1^. O’Brien *et al.* deposited Pd-Ag films onto a γ-Al_2_O_3_ (5–20-nm pore size) using DC magnetron sputtering [15]. Pd-Ag films were prepared with a thickness ranging from 0.7–1.1 µm at a 0.05-µm·min^−1^ deposition rate by changing the substrate bias and the substrate temperature (by external heating). At room temperature and 1 bar pressure, the samples were N_2_ permeable, and the gas flow ranged from 8 × 10^−9^–1 × 10^−6^ mol·m^−2^·s^−1^·Pa^−1^ depending on the deposition parameters (mainly RF bias). The sample analysis by SEM revealed that the coatings contained pinholes, and their number and size mostly depended on the substrate conditions before deposition. Hoang *et al.* deposited 0.75 µm-thick Pd_77_Cu_23_ alloy films by DC sputtering using pure Pd and Cu targets independently controlled [16]; XRD analysis showed that the deposited film has a polycrystalline structure with a grain size of 14 nm, when the substrate temperature was 300 K and 240 nm with a substrate temperature of 600 K; the grain size of the film deposited at 400 K is about 60 nm according to SEM surface analysis. During the last few years, SINTEF studied the growth of Pd-based films onto polished silicon substrates by AFM; in a very recent study, they found that the roughness of the Pd_77_Ag_23_ films increases from 8.4–13.2 nm when increasing the film thickness from 2.2–11.2 µm [11]. In a previous work, the same group also determined the grain size by TEM analysis; the region near the silicon substrate presented small grains of 12–18 nm, and when growing a 5 µm-thick layer, the grain size increased up to 100 nm. Thus, the larger the grains present in the film, the rougher the surface, as also stated by Zhang *et al.* [17]. Recently, Pereira *et al.* [18] prepared Pd-Ag films (0.7–1.4-µm thick) by PVD-MS onto yttria-stabilized zirconia (YSZ)-γ-Al_2_O_3_ (5-nm pore size) top layer support using two separate targets of Pd and Ag and at a substrate temperature of 473 K. The N_2_ permeance of these membranes ranged from 8 × 10^−8^–9 × 10^−7^ mol·m^−2^·s^−1^·Pa^−1^ at 200 kPa and room temperature depending on the Pd-Ag film thickness. On the other hand, some recent research showed that the microstructure and morphology of Pd-based layers (e.g., grain size, grain boundary effect) may play an important role in the hydrogen separation properties [19,20,21]. It is obvious that different membrane microstructures are obtained by using different preparation techniques (ELP, PVD, *etc.*) and/or process parameters (e.g., temperature, deposition rate, post-treatments, *etc.*).

The aim of this work is to study the effect of the substrate temperature on the roughness and surface morphology phenomena, in ultra-thin Pd_77_Ag_23_ layers (≤2 µm thick) grown by direct PVD-MS deposition onto polished Si wafers (the smoothest surface available), the effect of the support on the layer growth being negligible. The grain size, density and roughness evolution of 0.6 µm-thick Pd-Ag layers deposited on Si wafers have been experimentally obtained as a function of substrate temperature by atomic force microscopy (AFM), as well as the main physical processes governing the film morphology. AFM has not been used before for measuring the grain size (lateral correlations extracted by PSD function), roughness and density of Pd-based layers, and it gives valuable information to improve our understanding of the growth process and to optimize the PVD process parameters. Subsequently, 0.5–2 µm-thick Pd-Ag layers have been deposited onto commercially-available asymmetric porous ceramic tubes to study the influence of the porous support on the Pd-Ag layer microstructure. The characterization of these supported layers has been completed by XRD, SEM and nitrogen permeation analysis. The obtained SEM images of the supported layers have been compared to Thornton’s model of the structure zone. The production of an optimized membrane (in terms of flux and or selectivity) is outside the scope of the present paper.

## 2. Results and Discussion

### 2.1. Influence of the Heating Power on the Morphology Evolution

Grain size is an important parameter to consider in the preparation of dense Pd-Ag membranes due to the possibility of defect formation in the space created between the grains. This is reflected in the hydrogen permeation properties of Pd-Ag thin films producing membranes with low hydrogen selectivity, as reported previously [19,20,21]. On the other hand, the grain size increases when increasing the substrate temperature [22]. The grain size depends also on several factors, such as substrate material, film thickness, magnetron size and external substrate heating during deposition. Hence, the aim of this work is to study the influence of the process parameters on the grain size of the film. The influence of the power applied to the heaters (directly proportional to the coating temperature) on the surface morphology evolution during the layer growth onto Si (001) wafers was studied by AFM. Four heating power values were applied: 0, 500, 1000, 1500 W. The operating temperature for each process was measured using temperature indicators (stickers) and is listed in Table 2, together with the deposition times and rates.

**Figure 1:**
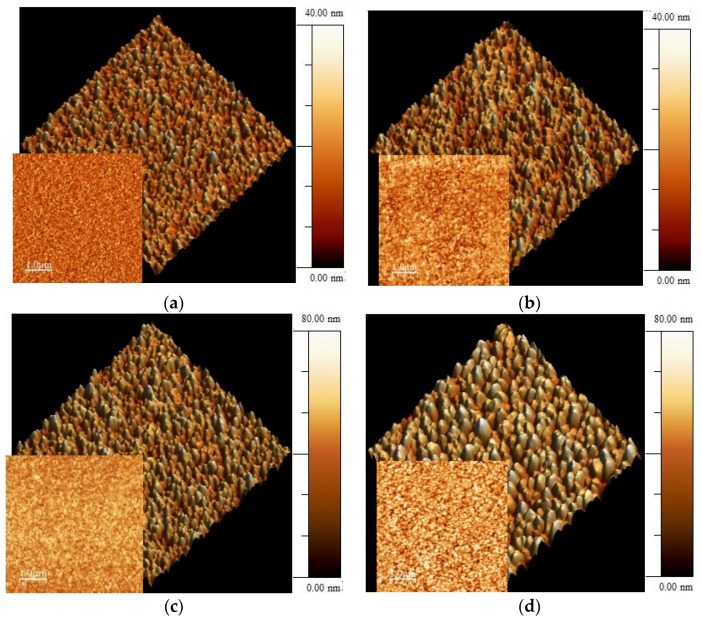
The 2 × 2 µm^2^ (and 5 × 5 µm^2^ smaller image in the left bottom part) tapping mode AFM images of the Pd-Ag films grown at different heating powers applied to substrate holder (measured temperature using stickers): (**a**) 0 W (<363 K); (**b**) 500 W (405–410 K); (**c**) 1000 W (497–506 K); (**d**) 1500 W (506–515 K). A lateral bar showing the difference in height between the lowest and the highest points is included for each case.

Figure 1 shows representative AFM images of 0.6 µm thick Pd-Ag films grown on Si (001) surfaces at various temperatures. For temperatures lower than 430 K (heating powers of 500 and 0 W), a layered morphology displaying featureless structures has been obtained (Figure 1a,b) in which the grains do not aggregate into larger structures. However, for the films grown at 505–515 K (1500 W heating power), larger structures were observed (250–270 nm wide) due to the grain cluster formation (Figure 1d). A similar structure was obtained for the layer grown at 1000 W of heating power (495–505 K), although it is not clearly visible in Figure 1c.

**Figure 2:**
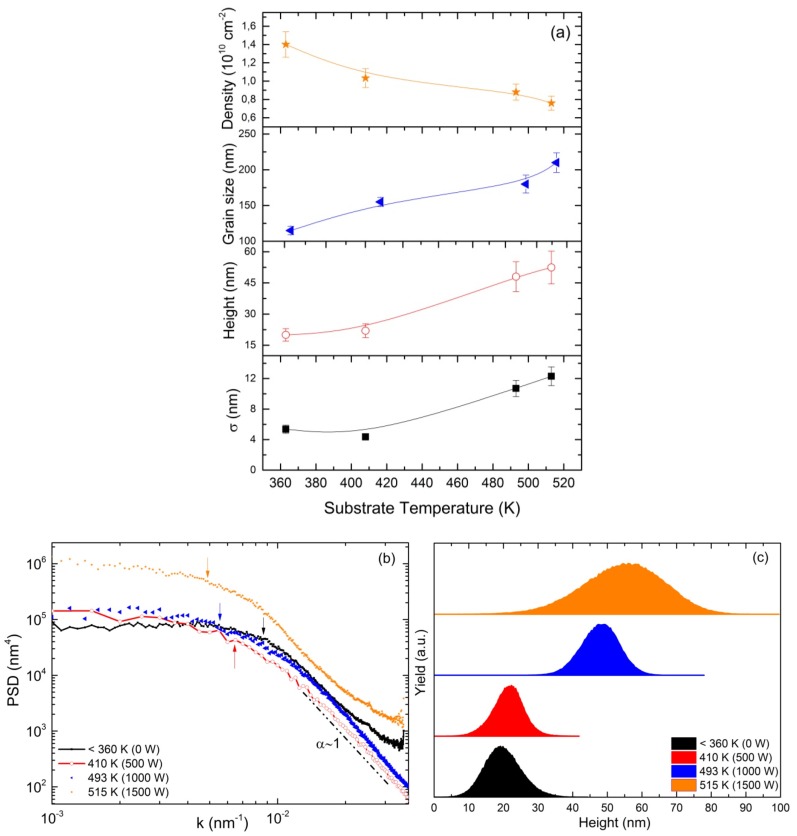
(**a**) The surface roughness σ, height, grain size and density (from bottom to top) obtained from PSD curves; (**b**) PSD *versus* *k*; and (**c**) the surface height obtained from the AFM data for 0.6 µm-thick Pd-Ag films grown on Si (001) at different heating powers (or substrate temperatures).

The analysis of these data is shown in terms of roughness σ, height, grain size and density (Figure 2a), PSD (Figure 2b) and height histogram (Figure 2c) as a function of the temperature of the substrate. From Figure 2a, a sharp transition in the 430–490 K range in the surface characteristics is observed. By reducing the temperature, the film roughness (σ) reaches values close to 4 nm, heights around 20 nm and a grain size (extracted from the PSD curves) close to 120 nm. At higher temperatures, these roughness parameters reach higher values. Further insight into the growth mechanisms involved in each case can be obtained from the analysis of the PSD curves (Figure 2b). Films deposited at different heating power values (0 W, 500 W and 1000 W) display an initial scaling region dominated by the surface diffusion relaxation process (α ≈ 1). The saturation of the PSD curve, where the slope changes from almost zero (*i.e.*, it runs parallel to the *x*-axis) to negative values, provides the grain size of the Pd-Ag films. The PSD almost saturates for *k* <0.01 nm^−1^, which corresponds to the average lateral grain size of *l* >100 nm. [23]. Thus, at T < 410 K (0 W and 500 W), the PSD curve is shifted downwards, which implies a smoother surface. For the films grown at 1500 W of heating power (the highest temperature in this work), the PSD saturates when *k* reaches a value close to the inverse of ~240 nm (k ~0.004 nm^−1^), which is the average lateral value of the structures (Figure 2b). Besides, films grown at higher temperatures (>510 K) display also an initial linear scaling region in the logarithmic plot for *k >* 0.018 nm^−1^ (or 55 nm) consistent with α = 0.35 due to surface diffusion effects followed by an unstable regime. Moreover, this value of α = 0.35 is consistent with a non-linear hydrodynamic Kardar–Parisi–Zhang equation (KPZ) [24]. Changes in the slopes of the linear regime (where α changes from one to 0.35) mean different grain sizes appeared in the AFM image. In any case, in order to provide a full description of the grown system (model), it is important to find out the origin of the increased surface roughness and, accordingly, the surface slope. This type of analysis has not been reported before for Pd-based layers. The origin of the KPZ scaling in the experimental results can be understood as a complex dynamic growth of grains, which is dictated by an interplay between inter-grain surface roughness (yielded by the coalescence of neighboring grains) and surface diffusion processes by the temperature increase. This leads to a complex packing of crystalline grains.

**Figure 3:**
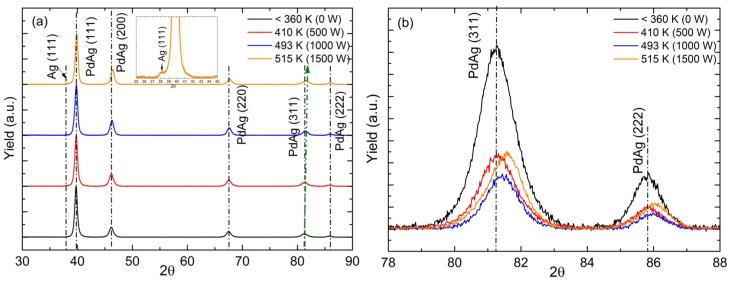
X-ray diffraction patterns of Pd-Ag layers deposited onto Si (001) at different substrate temperatures during the coating: (**a**) diffraction pattern between 2θ = 30°–90°, including a zoom in the 35-45 region; (**b**) zoom around 2θ = 78°–88° in order to observe more clearly the displacement of the maximum peak position of Pd-Ag (related to different Ag contents).

According to the XRD analysis shown in Figure 3, when increasing the heating power, the Pd-Ag alloy diffraction peaks shifted to the right (between the Pd and Ag reflections, which is characteristic of the lattice spacing of the fcc Pd-Ag alloy) having a lower Ag content and indicating that part of the silver was not alloyed. These Pd-Ag alloys, which have the face-centered cubic (fcc) phase, vary only in composition, but not in crystal structure. Ayrtuk *et al.* [25] discussed that the only effect of a change in the composition is the shift in the diffraction line positions in accordance with the change in the lattice parameter, which varies continuously from the pure Ag to the pure Pd. Therefore, it is possible to assume a linear relationship between the lattice constant of the alloy and the Ag content and to estimate the Ag content of the alloy [26]. For heating power of 0 W, a Ag content of ~19 wt.% was determined from the lattice constant of the Pd-Ag (*a* = 3.923 Å), which is lower than the Ag content of the target material. When increasing the heating power, the Ag content of the Pd-Ag alloy is reduced: the lowest Ag content of ~13 wt.% (*a* = 3.912Å) was obtained for the case with 1500 W of heating power, where a silver peak is clearly detected (Figure 3a) showing the part of the Ag that was not alloyed.

It was demonstrated by AFM and XRD results that for a heating power of 0 W, the Pd-Ag films presented the smallest grain size distribution and the closest Pd-Ag alloy to the 23 wt.% of the target. A smaller grain size gives a closer structure with less space between the grains (defects); therefore, a heating power of 0 W was chosen for the deposition on porous tubular supports.

### 2.2. Influence of the Support and Pd-Ag Layer Thickness

**Figure 4:**
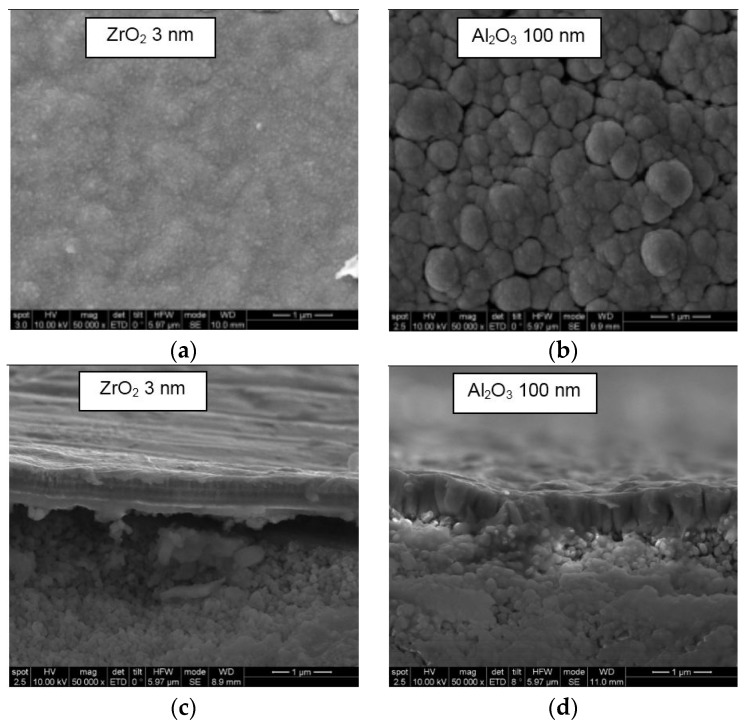
SEM images of the Pd-Ag layer (thickness: ≈0.5 µm) deposited on the outer surface of asymmetric tubes: ZrO_2_ 3 nm pore size (surface (**a**) and cross-section (**c**)) and Al_2_O_3_ 100 nm pore size (surface (**b**) and cross-section (**d**)).

Two micron-thick membranes were deposited onto Al_2_O_3_ 100 nm, ZrO_2_ 110 nm and ZrO_2_ 3 nm porous tubes (membrane length: 22–23 cm long) in the same process using a target power of 1500 W and a heating power of 0 W. Pd-Ag membrane layers deposited on ZrO_2_ 3 nm were completely delaminated, on ZrO_2_ 110 nm were partially delaminated and on Al_2_O_3_ 100 nm did not show any delamination. This delamination may occur due to the poor adhesion of the Pd-Ag layer and the stresses involved during the deposition and cooling processes. However, when analyzing the inner surface of the delaminated Pd-Ag layer, ZrO_2_ was found. Therefore, delamination between the top layer and the alumina base support could not be disregarded, as well. A similar delamination behavior was also reported before by Jayaraman *et al.* [12]. The N_2_ permeance of the 2 µm-thick layer onto Al_2_O_3_ 100 nm support was 1.4 × 10^−6^ mol·m^−2^·s^−1^·Pa^−1^ at 25 °C, which is much higher than the desired value for its use for hydrogen purification (<1 × 10^−9^ mol·m^−2^·s^−1^·Pa^−1^), while for the Al_2_O_3_ 100 nm support itself, the N_2_ permeance was 3.0 × 10^−5^ mol·m^−2^·s^−1^·Pa^−1^ at 25 °C. Afterward, the thickness of the Pd-Ag layer was reduced to 0.5 microns in order to analyze the effect of each support on the final quality of the membrane layer without being delaminated. The Pd-Ag layer growth onto ZrO_2_ 3-nm and Al_2_O_3_ 100 nm pore size tubes was studied. Figure 4 shows the surface SEM images of the resulting Pd-Ag layers. The quality of the final PVD Pd-Ag layer considerably depends on the pore size of the support. As can be seen in Figure 4a,c, the Pd-Ag layer itself has a really close columnar structure (without visible open pores) when it is deposited onto ZrO_2_ 3-nm porous support (small pore size). However, if the Pd-Ag layer is deposited with the same conditions onto an Al_2_O_3_ 100 nm porous support, it presents an open structure with a more heterogeneous columnar growth (see Figure 4b,d). For the next part of the study, the ZrO_2_ 3-nm support was chosen to prepare thinner Pd-Ag films.

### 2.3. Influence of the Target Power and Pd-Ag Layer Thickness

The sputtering technique is characterized for producing smooth surfaces with low values on surface roughness when using specular substrate surfaces, like polished silicon wafers. However, in rougher substrates, the sputtering technique reproduces the surface roughness, leading to different structure growth. When increasing the substrate temperature, an increase in the surface mobility is produced, causing a smoother surface on the films by the filling of the concavities [22]. However, an exception is the special case where the deposited material has a tendency to grow preferentially along the determined crystalline phase because of either large anisotropy in the surface energy or by the presence of faceted roughness on the substrate, as is the case when using porous alumina supports.

**Figure 5:**
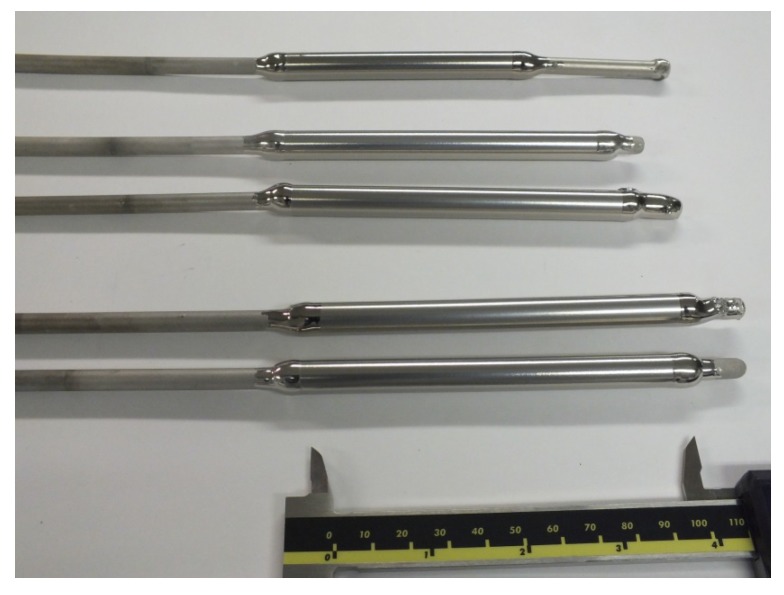
The ≤1 µm-thick Pd-Ag-supported membranes onto ZrO_2_ 3-nm tubular supports.

The influence of the target power and thickness on the final quality of the Pd-Ag layer on 8–10 cm long ZrO_2_ (3 nm pore size) porous support was studied. The target powers used were 350, 1500 and 3000 W; the heating power during the coating was set to 0 W; and the deposited layer thicknesses were 0.5 and 1 µm. In these cases, no delamination was observed (see Figure 5).

**Figure 6:**
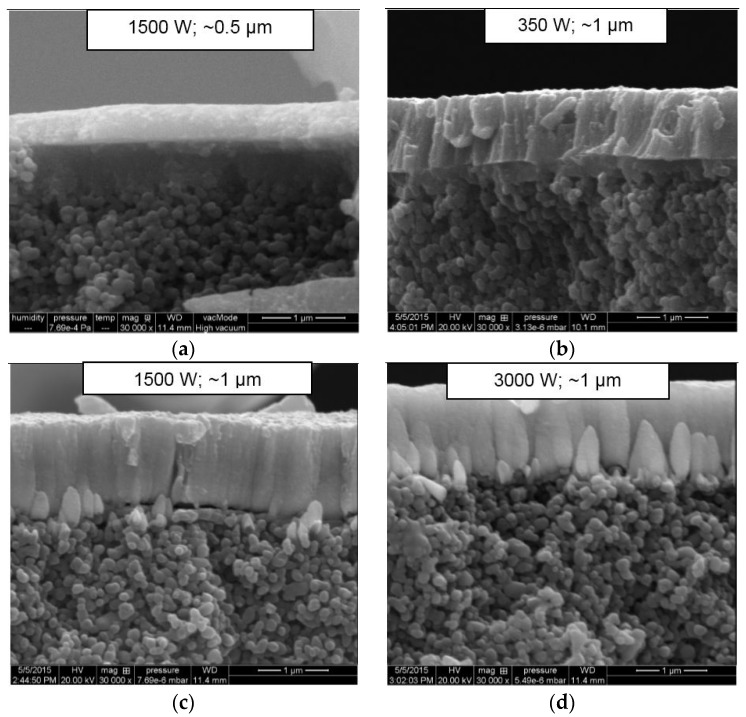
SEM cross-section images of Pd-Ag layers with different target powers and membrane thicknesses: (**a**) 1500 W; 0.5 µm; (**b**) 350 W; 1 µm; (**c**) 1500 W; 1 µm; and (**d**) 3000 W; 1 µm.

**Figure 7:**
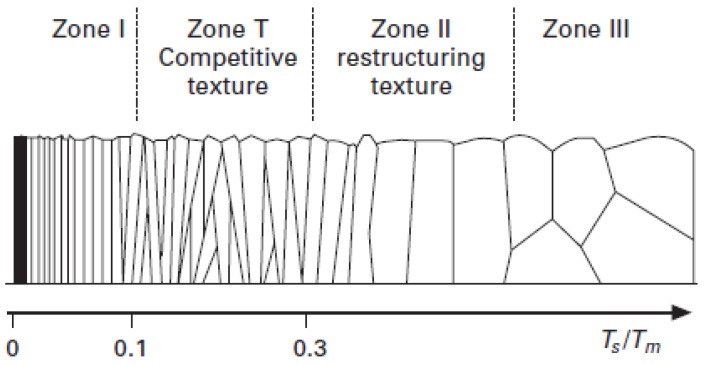
Main characteristics of the structure zones of Barna and Adamik’s model [29], which is an update of Thornton’s model.

Figure 6 shows the SEM cross-section images of the Pd-Ag layers prepared with different target powers and membrane thicknesses deposited onto the ZrO_2_ 3 nm porous tube. The structure of the layer deposited at 3000 W was related to the structures reported by Thornton’s model [27], in which the morphology of a grown layer is compared with the ratio between the temperature of the substrate (Ts) and the melting point of the material (Tm, which is 1523 K for Pd_75_Ag_25_ [28]), as shown in Figure 7. At 3000 W of target power (*Ts*/*Tm* = 0.27), the Pd-Ag layer presents a V-shaped columnar structure that is representative of competitive growth, called Zone T (0.1 < *T*/*Tf* < 0.3) [27]. Pereira *et al.* [18] obtained a similar morphology for a 0.7 micron-thick Pd-Ag layer deposited at a substrate temperature of 473 K on nanoporous YSZ/γ-Al_2_O_3_. The columns are characteristic of the result of low atoms mobility, which is directly related to a low substrate temperature.

The N_2_ permeances at 298 K of the prepared layers as a function of the target power and thickness of the deposited layers are presented in Table 1. At the same heating power (1500 W), when the membrane thickness increases from 0.5–1 µm, the N_2_ permeance is reduced as expected. Among the 1 µm thick membranes, there is a good correlation between the increase of heating power and the increase in N_2_ permeance. It should be noted that, since the lowest N_2_ permeance value obtained (1.3 × 10^−6^ mol·m^−2^·s^−1^·Pa^−1^) is much higher than the desired value (<1 × 10^−9^ mol·m^−2^·s^−1^·Pa^−1^), these Pd-Ag membranes are not suitable for high H_2_ purity applications.

**Table 1:** N_2_ permeance at 25 °C and 1 bar pressure difference of the Al_2_O_3_ 100 nm and ZrO_2_ 3 nm support tubes and the Pd-Ag PVD layers deposited onto these support varying the target power (300–400, 1500, 3000 W) and the thicknesses (0.5, 1.0, 2.0 µm).

Sample	N_2_ Permeance × 10^−9^ (mol·m^−2^·s^−1^·Pa^−1^)
ZrO_2_ 110 nm (support)	35,500
Al_2_O_3_ 100 nm (support)	30,300
ZrO_2_ 3 nm (support)	24,600
Pd-Ag layer (1500 W, 2.0 µm) on Al_2_O_3_ 100 nm	1400
Pd-Ag layer (1500 W, 2.0 µm) on ZrO_2_ 110 nm	Delaminated
Pd-Ag layer (1500 W, 2.0 µm) on ZrO_2_ 3 nm	Delaminated
Pd-Ag layer (1500 W, 0.5 µm) on ZrO_2_ 3 nm	15,143
Pd-Ag layer (350 W, 1.0 µm) on ZrO_2_ 3 nm	1288
Pd-Ag layer (1500 W, 1.0 µm) on ZrO_2_ 3 nm	2297
Pd-Ag layer (3000 W, 1.0 µm) on ZrO_2_ 3 nm	3974

The N_2_ permeance values differ in some cases to the ones related to the state-of-the-art membranes, because they depend on the type and quality of the support, layer thickness and PVD process conditions (mainly temperature) used in each case, but the N_2_ permeances reported in the state of the art were also above the desired value. An approach to increase the low selectivity of Pd-based layers by PVD is to deposit a Pd-based layer by ELP on top, as reported by the authors [30]. Dittmar *et al.* used a PVD layer as the activation step for membranes prepared by ELP and EP [31]. PVD can also be used for adding another metal to a Pd-based layer prepared by ELP in order to adjust the composition of the final alloy [32].

## 3. Materials and Methods

### 3.1. Membrane Preparation

**Figure 8:**
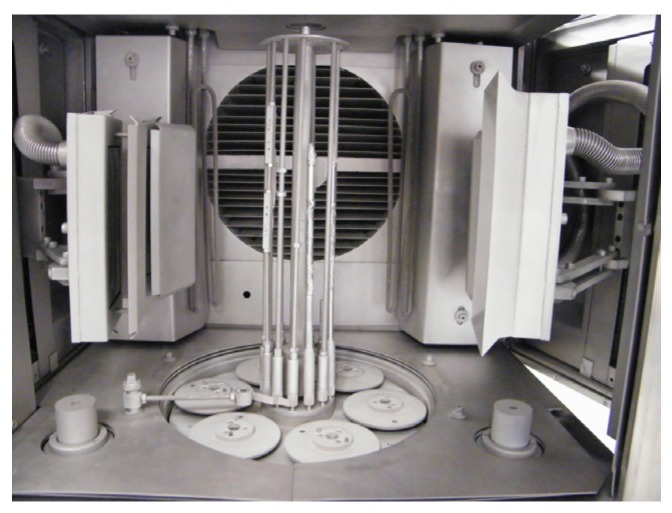
Internal view of the CemeCon^®^ CC800/8 PVD equipment.

Ultra-thin Pd-Ag layers (0.5–2 µm thick) were deposited by PVD magnetron sputtering on tubular porous alumina supports (10/7 mm o.d./i.d.) with different top layers (pore size) (provided by Rauschert Kloster Veilsdorf, Veilsdorf, Germany) and polished silicon wafers (purchased from Siegert Wafer GmbH , Aachen, Germany). The material and pore size of the top layers of the alumina tubes were Al_2_O_3_ 100 nm, ZrO_2_ 110 nm and ZrO_2_ 3 nm. Polished silicon (001) substrates were used as supports just to study the microstructural evolution and not as membrane support. Commercial direct current (DC) magnetron sputtering equipment (CemeCon^®^ CC800/8, Würselen, Germany) with four rectangular targets (200-mm height × 88-mm width × 5-mm depth) of Pd-Ag alloy (23% Ag) was used in this work. In Figure 8, a picture of the inside of the PVD chamber is shown, including the positioning of the targets and supports. The heaters are placed in the internal part of the chamber’s front door. For proper handling of the membranes, prior to Pd-Ag layer deposition, the ceramic porous tubes were joined to two dense ceramic tubes using a glass sealant. Before each deposition process, the sputtering chamber was evacuated to base pressure lower than ~1 × 10^−5^ Pa and then back-filled with ultra high purity (UHP) argon at a pressure of 500 mPa. The target to substrate distance was held constant at 80 mm for all processes. For each process, the sample holder was negatively charged at −140 V. Two different experiments were carried out: (i) heating of the substrate holder; and (ii) changing the power applied to the Pd-Ag targets. In the first case, the heating power was varied for each process at 0 W, 500 W, 1000 W and 1500 W (see Table 2). For the second series of experiments, the DC target power for layer deposition was varied for each process with 350 W, 1500 W and 3000 W, respectively. The temperature ranged from near room temperature to 515 K due to the plasma heating effect and the power applied on the heaters. The temperature during the coating step was measured with self-adhesive temperature labels attached to the sample holder (substrate temperature). The thickness of Pd-Ag alloy film was controlled by changing the sputtering time (see Table 2), and before each process, the deposition rate was determined by performing at least two sputtering processes with different times and measuring the thickness of the grown layers by using a Veeco DEKTAK 150 contact profilometer between the coated and uncoated surface of the same sample. These preliminary sputtering processes coated the internal walls of the chamber of Pd-Ag and avoided the deposition of any other element from the chamber to the membrane.

**Table 2:** Process parameters applied in each PVD process.

Heating Power (W)	Target Power (W)	Deposition Time (min)	Deposition Rate (µm·min^−1^)	Substrate Temperature (K)
0	1500	15	0.04	<360
500				405–410
1000				495–505
1500				506–515
0	350	80	0.01	NA
	1500	16	0.06	NA
	3000	8	0.13	405–433

NA: not available.

### 3.2. Membrane Characterization

The surface morphology was analyzed by atomic force microscopy (AFM), and the cross-section of the prepared samples was analyzed by an FEI Inspect F50 SEM-EDX in order to determine the structure and thickness of the Pd-Ag layer.

AFM measurements were performed in air, operating in taping mode with silicon cantilevers (nominal radius of 10 nm) with the Nanoscope IIIa equipment (Veeco, Freemont, CA, USA). The AFM images power spectral density (PSD) curves and the surface roughness were obtained by using the software of the equipment (Nanoscope Analysis v.3.1.2, Bruker, Billerica, MA, USA). The PSD curves were used to determine the grain size of the prepared Pd-Ag layers onto silicon wafers. The PSD function is defined as $PSD\left(k\right)=\frac{4\pi^{2}}{L^{2}}\langle H\left(k\right)H\left(-k\right)\rangle$, where *H(k)* is the 2D Fourier transform of the surface height $h\left(r\right)-\bar{h}$ in a system of lateral size *L*, and *k* is the wave-vector of modulus k= |k| [33,34]. For a rough surface, the PSD function behaves generically as $PSD\;\propto \;k^{-2\left(\alpha +1\right)}$ [35]; hence, from a logarithmic plot of the PSD *vs.* *k*, it is possible to calculate the value of the roughness exponent α at the corresponding length scales [36]. The α value provides information on the layer growth mechanisms, and therefore, it is possible to study the microstructure of the grown layer (e.g., grain size). The flooding tool in the AFM software analysis was used for measuring the density of the grains. The option “view perimeters from hills” was selected, in which the height was chosen according to the maximum height of grains, avoiding measuring irregular forms.

The composition of each membrane was examined by an X-ray diffractometer (Bruker D8 Advance diffractometer, with Cu K_α_ radiation at 40 kV and 40 mA). The scan rate was 0.02 °/s in the range 2θ = 30°–90° and in a grazing incidence mode (θ*_i_* = 2°). The N_2_ permeance (gas tightness) of the prepared Pd-Ag-supported samples was characterized at room temperature and 1 bar of pressure difference in membrane permeation test equipment. A shell and tube module configuration was used to test the membranes [37]. The N_2_ enters through the shell side, and permeate flow is measured with a HORIBA film flow meter using two different measuring tubes: VP-3U (20–1000 mL/min) and VP-4U (0.2–10 L/min). The pressure in the shell side is controlled by a back pressure regulator installed in the retentate side.

## 4. Conclusions

Thin Pd-Ag films have been deposited by PVD magnetron sputtering onto polished silicon sheets and porous ceramic supports. The effects of the coating temperature (heating power, target power), substrate pore size and film thickness on the morphology and gas-tightness of the coated Pd-Ag films have been studied. With a heating power of 0 W, the layers grown onto Si presented the smallest grain size distribution (~120 nm) and the closest Pd-Ag alloy composition (~19%) to the desired 23 wt.% of the used target. When increasing the heating power (temperature), AFM studies indicate that the surface roughness and grain size of the Pd-Ag layer increased, and the Ag content in the Pd-Ag film decreased (the lowest Ag content of ~13 wt.% was found at 1500 W of heating power). For the first time for Pd-based films, growth mechanisms have been identified from the results obtained by AFM analysis. At temperatures up to 500 K, the Pd-Ag film growth is governed by surface diffusion mechanisms (α ≈ 1 for *k >* 0.01 nm^−1^), but at temperatures above 510 K, the film growth could correspond to a KPZ (α = 0.35) behavior, leading to a complex packing of crystalline grains. On the other hand, when depositing a Pd-Ag layer by PVD onto a porous support, a closer structure was obtained when using a smaller pore size support (3-nm pore size compared to 100 nm). Increasing the film thickness (e.g., from 0.5–1 µm) favors the gas-tightness of the coated film supported on ZrO_2_ 3-nm porous support (smallest available pore size). However, thick films (e.g., 2 µm) may delaminate from the support due to poor adhesion and stresses involved during the deposition and subsequent cooling step and/or the delamination of the ceramic top layer from the base support. The change in the target power (that is linked to the substrate temperature) also affects the layer structure. All of the Pd-Ag layers deposited onto ZrO_2_ 3-nm supports at 0 W heating power with a thickness up to 1 µm presented a high N_2_ permeance at room temperature (from 1.3 × 10^−6^–3.9 × 10^−6^ mol·m^−2^·s^−1^·Pa^−1^); Thus, these ultra-thin Pd-Ag films directly deposited by PVD onto porous supports are not directly suitable for high purity hydrogen separation.
